# Medication Prescribed Within 1 Year Preceding Fall-Related Injuries in Older Adults in Ontario, Canada

**DOI:** 10.1093/geroni/igaa057.761

**Published:** 2020-12-16

**Authors:** Yu Ming, Aleksandra Zecevic, Richard Booth, Susan Hunter, Andrew Johnson, Rommel Tirona

**Affiliations:** 1 Western University, London, Ontario, Canada; 2 Western University, London, ON, London, Ontario, Canada

## Abstract

Background: The consequences of fall-related injuries are becoming more significant due to ageing societies worldwide. This study aims to provide information on medications prescribed to older adults within one year before they experienced fall-related injury in Ontario, Canada. Methods: A population-based descriptive study of older adults (66 years and older) who experienced fall-related injury was conducted using administrative secondary health care data of Ontario. The percentages of patients prescribed each Anatomical Therapeutic Chemical 4th level medication class and fall-risk increasing drugs one year before their fall-related injuries was summarized. Results: From 2010 to 2014, 288,251 older adults (63.2% females) were admitted to Emergency Department due to fall-related injury, 39.9% were fall-related fractures, 12.6% were head injuries. One year prior to their injury, 48.46% of older adults were prescribed with statins; 35.23% were prescribed with diuretics; 26.84% were prescribed with antidepressants; 25.90% were prescribed with opioids and 16.61% were prescribed with anxiolytics. A higher percentage of females were prescribed with diuretics, antidepressants, and anxiolytics than males. 85 years and older people had higher percentage of prescription of diuretics, antidepressants and antipsychotics than other age group. Discussion: In general, older adults diagnosed with fall-related injuries were prescribed with more opioids, benzodiazepines and antidepressants than other general older adults. There were distinct patterns of prescription medication within each sex and age group (66-74 group, 75-84 group and 85 years and older group). Further association between medications and fall-related injuries need to be established using well-defined cohort studies.

